# Morphometry, Bite-Force, and Paleobiology of the Late Miocene Caiman *Purussaurus brasiliensis*

**DOI:** 10.1371/journal.pone.0117944

**Published:** 2015-02-17

**Authors:** Tito Aureliano, Aline M. Ghilardi, Edson Guilherme, Jonas P. Souza-Filho, Mauro Cavalcanti, Douglas Riff

**Affiliations:** 1 Departamento de Geologia, Universidade Federal de Pernambuco, CEP 50740-530, Recife, Pernambuco, Brazil; 2 Departamento de Geologia, Universidade Federal do Rio de Janeiro, CEP 21949-900, Rio de Janeiro, Rio de Janeiro, Brazil; 3 Laboratório de Pesquisas Paleontológicas, Universidade Federal do Acre, CEP 69915-900, Rio Branco, Acre, Brazil; 4 Ecoinformatics Studio, P. O. Box 46521, CEP 20551-970, Rio de Janeiro, Rio de Janeiro, Brazil; 5 Instituto de Biologia, Universidade Federal de Uberlândia, CEP 38400-902, Uberlândia, Minas Gerais, Brazil

## Abstract

*Purussaurus brasiliensis* thrived in the northwestern portion of South America during the Late Miocene. Although substantial material has been recovered since its early discovery, this fossil crocodilian can still be considered as very poorly understood. In the present work, we used regression equations based on modern crocodilians to present novel details about the morphometry, bite-force and paleobiology of this species. According to our results, an adult *Purussaurus brasiliensis* was estimated to reach around 12.5 m in length, weighing around 8.4 metric tons, with a mean daily food intake of 40.6 kg. It was capable of generating sustained bite forces of 69,000 N (around 7 metric tons-force). The extreme size and strength reached by this animal seems to have allowed it to include a wide range of prey in its diet, making it a top predator in its ecosystem. As an adult, it would have preyed upon large to very large vertebrates, and, being unmatched by any other carnivore, it avoided competition. The evolution of a large body size granted *P. brasiliensis* many advantages, but it may also have led to its vulnerability. The constantly changing environment on a large geological scale may have reduced its long-term survival, favoring smaller species more resilient to ecological shifts.

## Introduction

The extinct genus of the giant caiman *Purussaurus* (Crocodylia: Alligatoridae: Caimaninae) thrived in the northern part of South America, or Pan-Amazonia, during the Middle to Late Miocene. Throughout this period, huge drainage basins, known as the Pebas and Acre mega-wetland systems, were established in the area. These expanses made up a complex system of deltaic, estuarine, swamp, and fluvial environments, which, in multiple macro-habitats, supported a remarkably rich biota [1, 2]. A diverse and widespread crocodilian assembly existed until the demise of such wetlands in the Pliocene, when *Purussaurus* became extinct concomitant with the extinction or continuing poverty of many other groups, all as large to giant (e.g.: gharials and nettosuchids) crocodilians, as well as small and specialized caimanines, large eupleurodiran turtles, several mammals, fish, molluscs and ostracods [3, 4, 5, 6]. This depauperisation of the aquatic and riparian fauna in northern South America followed the onset of the modern Amazon River system in the Pliocene, the most dramatic Amazonian change driven by faster and more extensive Andean mountain building, between Latest Miocene and Pliocene, 7–2.6 My [2].

Three described species of *Purussaurus* are known to have roamed these ancient wetlands: *P*. *neivensis*, from the Middle Miocene La Venta Formation (Colombia) [7, 8]; *P*. *mirandai*, from the Upper Miocene Urumaco Formation (Venezuela) [9]; and the largest one, *P*. *brasiliensis* from the Upper Miocene Solimões Formation (Brazil) [10, 11, 12, 13]. Materials associated with *P*. *brasiliensis* have also been found in the Cobija Formation (Bolivia), correlated with the Upper Miocene Solimões Formation from Brazil [14, 15]. Several authors have mentioned yet more material with affinities to *Purussaurus* from the Middle Miocene Ipururo Formation (Peru) [4, 16], but it has not yet been associated with any other formerly described *Purussaurus* species.

**Fig 1:**
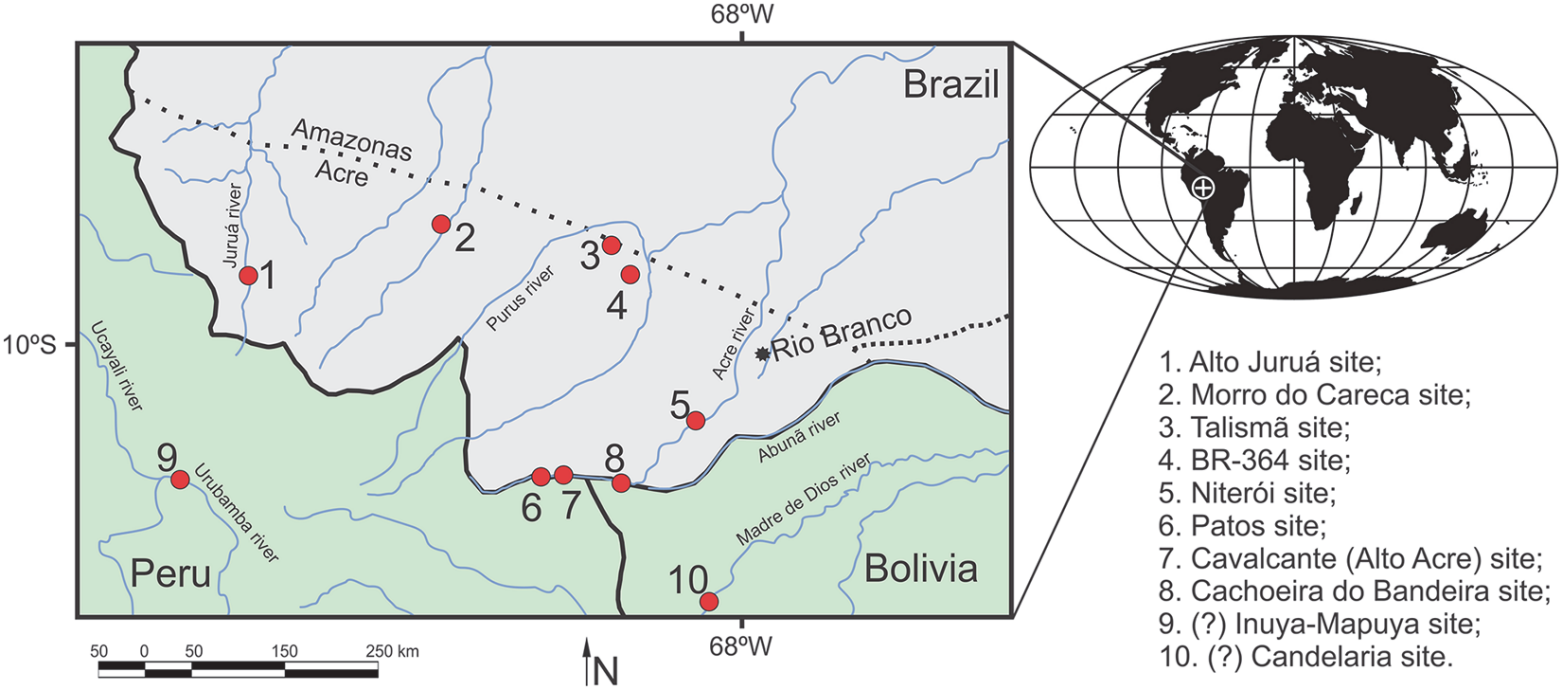
Late Miocene fossil sites in Southwest Amazonia. *P*. *brasiliensis* specimens recovered from sites 1–8. More specimens encountered at the Peruvian and Bolivian sites were assigned to *Purussaurus* sp. with no further taxonomic details. On the top right, the paleogeographical map showing the location of South America and the area of the Solimões Formation (white cross) during the Late Miocene (about 8 million years ago). Mollweide projection, latitude and longitude lines at 30° intervals. This map was created based on the work of Ron Blakey, available at http://cpgeosystems.com/paleomaps.html.

The object of the present analysis, *P*. *brasiliensis*, is known from several specimens found in erosive margins at low river levels, mainly along the Purus, Acre and Juruá rivers, the most complete specimen (UFAC 1403) being collected at Alto Acre site, in the municipality of Assis Brasil (Fig. 1).

**Fig 2:**
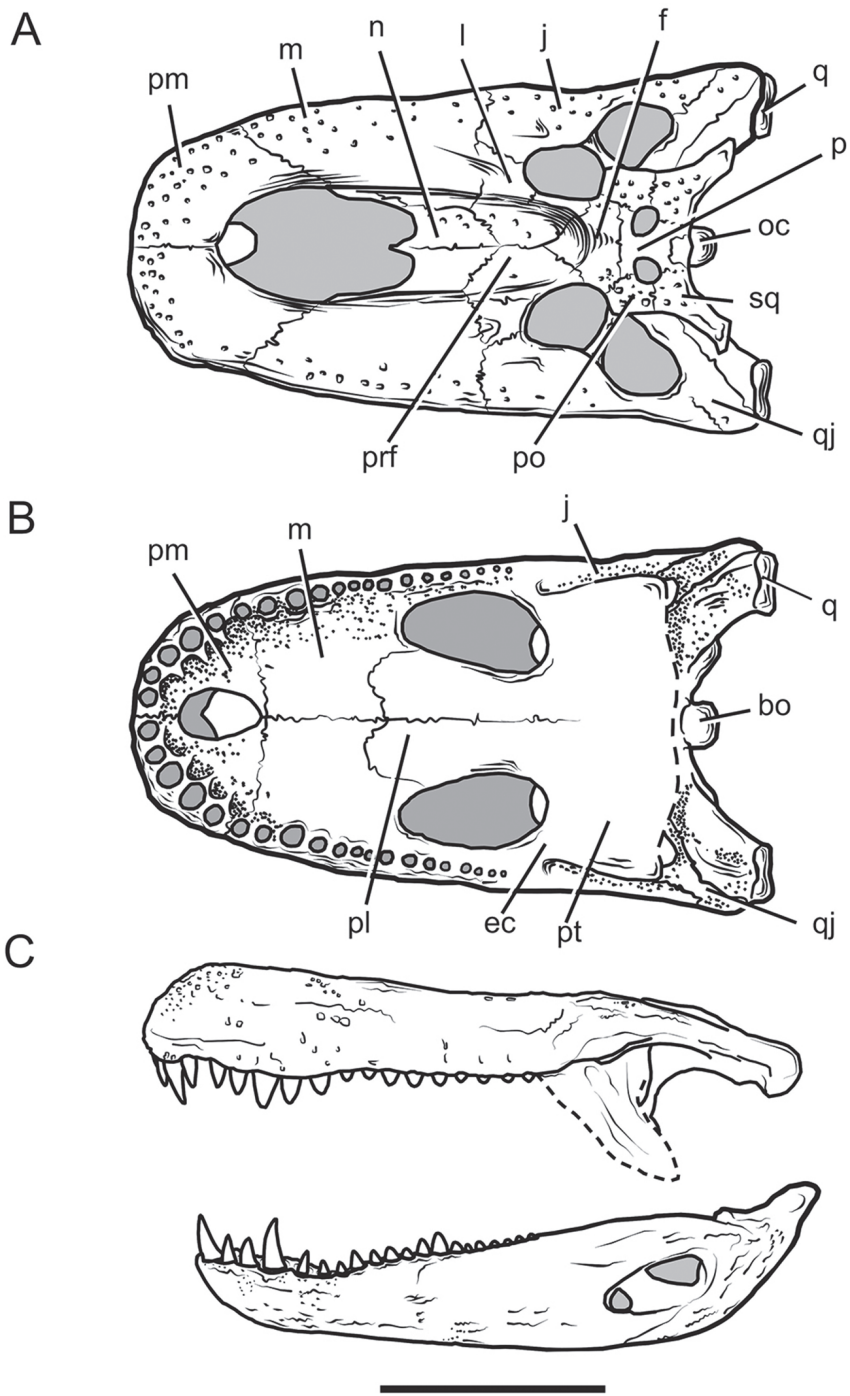
*Purussaurus brasiliensis* skull anatomy. (A) *P*. *brasiliensis* UFAC 1403 skull in dorsal view. (B) *P*. *brasiliensis* UFAC 1403 skull in ventral view. (C) *P*. *brasiliensis* reconstruction skull UFAC 1403 and associated mandible UFAC 1118 with teeth in lateral view. Scale bar: 50 cm. Abbreviations: bo, basioccipital; ec, ectopterygoid; f, frontal; j, jugal; l, lacrimal; m, maxilla; n, nasal; oc, occipital condyle; p, parietal; pl, palatine; pm, premaxilla; po, postorbital; prf, prefrontal; pt, pterygoid; q, quadrate; qj, quadratojugal; sq, squamosal.

The huge external naris, which occupies almost half of the rostrum in *P*. *brasiliensis* and *P*. *mirandai*, is the most characteristic feature of the genus (Fig. 2). *P*. *neivensis*, which nasals are not retracted, have wide, though not very long, external naris. The adult skull length is large to huge (857 mm for *P*. *neivensis*, 1260 mm for *P*. *mirandai* and 1400 mm for *P*. *brasiliensis*). The skull possesses large caniniform anterior teeth, with a crown height of approximately 100 mm in *P*. *brasiliensis*. A mandible described in 1967 from Juruá River has a length of 1750 mm [17]. The total body length of *Purussaurus brasiliensis* was superficially estimated by previous authors as something between and 11 and 13 m [4, 17, 18], making it one of the largest ever crocodyliforms. However, despite being known since the nineteenth century, *P*. *brasiliensis* is still poorly understood. Previous authors have published short descriptions, some of which in science meeting proceedings, and much of the relevant data is still not formally available [8, 10, 11, 12, 13, 17, 19]. Four partial skulls are known for this species, one complete with the exception for the pterygoids (Fig. 2, B and C), at least four pairs of mandibles, many cervical and dorsal vertebrae, and isolated teeth and osteoderms.

Besides *P*. *brasiliensis*, the Upper Miocene Solimões Formation has supplied many other crocodyliformes. Among them are: *Caiman brevirostris*, *C*. *niteroiensis*, *Mourasuchus amazonensis*, *M*. *nativus*, the gharials *Gryposuchus jessei* and *Hesperogavialis*, and the Crocodylidae *Charactosuchus* (at least two species). Studies focused primarily on the paleoecology of this crocodyliform fauna are nonexistent, and particularities about their biology are also unknown. Nonetheless, some authors have drawn general conclusions about the “Miocene Optimum” for crocodyliformes in South America [4, 20].

In Crocodyliformes, body size measures (*e*.*g*., snout-vent length, total length, and body mass) are closely related to various physiological and ecological features [13, 19], and estimating size and weight of extinct species is an important key to understanding their role in ancient ecosystems [21, 22]. Based on this premise, our team has made an attempt to obtain estimates of body size, weight, and bite force of the extinct caiman *P*. *brasiliensis*, in order to discuss implications related to these parameters, such as feeding ecology, and likely structural and physiological constraints regarding the large body size.

Feeding ecology is one of the main features of an organism that can be affected by body size [23, 24]. Living crocodilians will take a variety of prey, depending on availability, body size, and ontogenetic stage of the individual [25, 26, 27]. To be able to discuss that further, besides comparisons with modern analogues, and an analysis on the information available for *Purussaurus* dentition, we have made an attempt to predict the mean food intake of *P*. *brasiliensis* using ecological models available in the literature.

## Materials and Methods

Institutional abbreviations: **DGM**, Divisão de Geologia e Mineralogia do Departamento Nacional de Produção Mineral, Rio de Janeiro, Brazil; **UFAC**, Universidade Federal do Acre, Rio Branco, Brazil; **UFRJ-DG**, Departamento de Geologia da Universidade Federal do Rio de Janeiro, Rio de Janeiro, Brazil.

This study based its assessments on equations obtained from biometric studies of the extant crocodyliform *Caiman latirostris* [28], due to the phylogenetic proximity of this taxon to *Purussaurus*, and from morphometric data available for all 23 living species of crocodilians [22]. The specimen UFAC 1403 was analyzed for this study. The Laboratório de Pesquisas Paleontológicas at the Federal University of Acre at Rio Branco (UFAC), Brazil, hosts the studied material. No permits were required because the study was based on a museum specimen, and this work involved no excavation or fossil collection.

### Estimating Total Length, Body Mass and Bite-Force of *P*. *brasiliensis*

Morphometric data based on living taxa are commonly used to determine skeletal dimensions and body mass of extinct crocodylomorphs [22, 29, 30]. In this work, we applied the same general methodology to predict the total length and body mass of *Purussaurus brasiliensis*.

**Fig 3:**
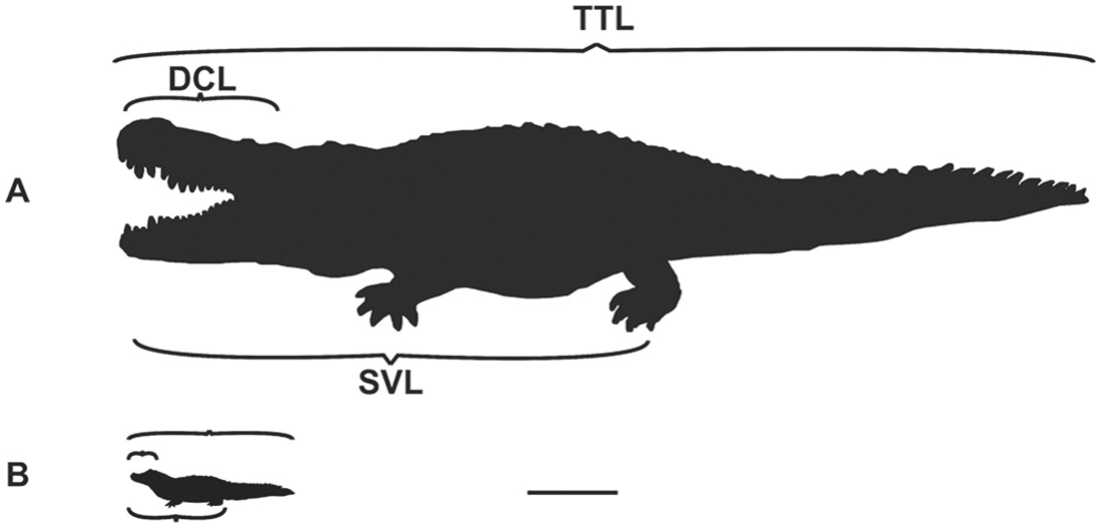
Measures and their abbreviations. DCL, skull length, SVL, snout-vent length, TTL, total length (A) *Purussaurus brasiliensis* (B) *Caiman latirostris*. Scale bar: 100 cm.

Biometric data obtained from *Caiman latirostris* [28] were applied to estimate its SVL and TTL (“Snout-Vent length” and “Total length”, in cm, respectively; see Fig. 3). Several morphological similarities with *P*. *brasiliensis* determined the choice of this related species, especially their phylogenetic relationship and body proportions [31, 32]. Statistical data obtained from *Alligator mississippiensis* individuals have shown that bite-force generation is statistically indistinguishable between same-sized individuals and that the BM (“Body Mass”, in kg) is the best measure to estimate an accurate bite strength [25, 32].

The fossil specimen used to access *P*. *brasiliensis*’ anatomical information was UFAC 1403 (described by [8], Fig. 2), held at the *Laboratório de Pesquisas Paleontológicas* collection at UFAC, Rio Branco, Acre, Brazil. It is an almost complete skull (DCL or “Skull length” = 1400 mm) with mandibles associated but pterygoid not preserved. It was an adult individual with no further information on sex. The other adult specimens (see Table 1) collected are too fragmentary and do not present the quality of preservation of the morphological characters required for the development of this study. UFAC 1403, although not the largest individual found so far, was chosen because of its completeness, as a key to access more accurate estimates.

**Table 1:** Known *Purussaurus brasiliensis* sincranium material (skull and mandibles).

Collection number	Material description	Literature
UFAC 1118	Complete mandible	Mentioned in [4]
UFAC 1403	Nearly complete skull; pterygoids and teeth are missing	Presented in a scientific meeting [3]
UFAC 4770	Fragmentary skull with posterior portion badly preserved	Mentioned in [4]
UFAC 5862	Fragmentary pair of mandibles with only the posterior portions preserved	Presented in a scientific meeting [17]
DGM 527-R	Fragmentary mandible with anterior portion preserved and teeth associated	Presented in a scientific meeting [16]
UFRJ-DG s/n (no number)	Fragmentary skull badly preserved still under preparation	Unpublished

We performed ordinary least-squares (OLS) regression analysis on the original data provided by [22] and [28] in order to estimate the values of SVL, TTL, BM and BF for *P*. *brasiliensis*. DCL was used to obtain the SVL. TTL was calculated based on the SVL. The TTL was applied to achieve BM, and finally, BM was used to obtain the BF (see Supporting Information for details). Since the aim of the analysis was to provide estimates of variables for the studied specimen, OLS was used instead of less conventional methods, such as reduced major axis [33, 34]. All data were log-transformed before analysis to homogenize the variances and provide a better fit to the allometric model. Confidence intervals and error estimation for the regression coefficients were computed using the bootstrap method [35, 36] with 1000 replications for each run. This method does not make any assumptions about the underlying distribution of the data and is well suited to the analysis of small data sets such as those typically encountered in paleontological studies [37]. All computations were performed using R version 3.03 [38] with the boot [39, 40] and simpleboot [41] packages. All the equations are presented in Table 1. See also Supporting Information.

### Estimating food intake of *P*. *brasiliensis*

Most research on feeding ecology of large crocodilian taxa such as *Melanosuchus niger*, *Crocodylus porosus*, and *C*. *niloticus* has been limited to juveniles and subadults. For large adults (individuals longer than 3 m) little quantitative data is available.

Hutton [42], however, studied the ecology of *C*. *niloticus*, collecting feeding data of several individuals of a variety of ages (from juveniles to very large adults) in different growth seasons during a three-year mark-recapture experiment. Hutton [42] was able to generate equations that predict the mean daily food intake for the growing season [Log_10_ [Croc BM / food intake] = 2.151] and non-growing season [Log_10_ [Croc BM / food intake] = 2.592] of *C*. *niloticus*. We used the same formulas, applying our estimate of BM to calculate the mean food intake of an adult *P*. *brasiliensis*.

## Results

**Fig 4:**
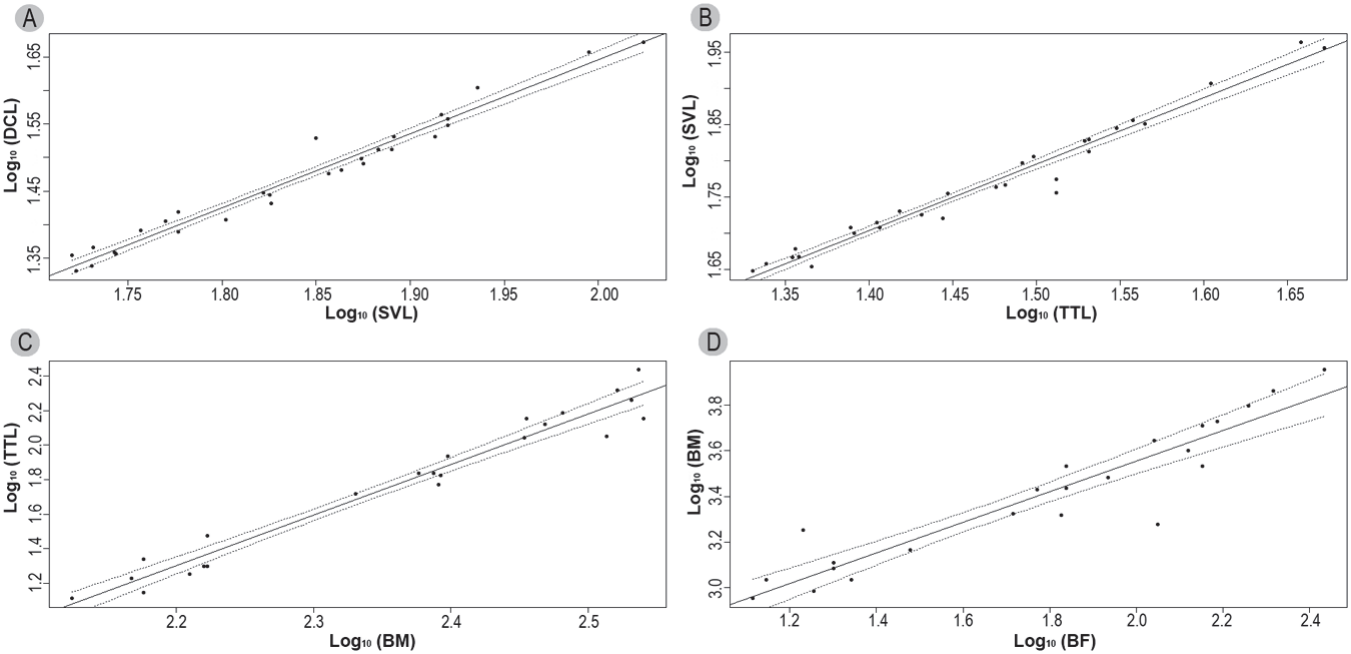
Regression lines with 95% confidence bands obtained by the bootstrap procedure of A, DCL versus SVL (data from [22]); B, SVL versus TTL (data from [22]); C, TTL versus BM (data from [20]); and D, BM versus BF (data from [20]).

The equations obtained from ordinary least squares regression analysis of the variables SVL, TTL, BM, and BF are presented in Table 2. The regression lines with 95% confidence bands by the bootstrap procedure for the same variables are displayed in Fig. 4. Size and mass estimates for *P*. *brasiliensis* calculated are shown in Table 3.

**Table 2:** Regression equations, with slope (a) and intercept (b), 95% confidence intervals (in parenthesis), bootstrap estimates of standard error (SE) and Pearson correlation coefficient (r).

Equation	a (CI)	SE	b (CI)	SE	r
Log_10_(SVL) = a + b * Log_10_(DCL)	-0.56913 (-0.71309, -0.42518)	0.0588	1.10776 (1.02959, 1.18592)	0.0322	0.9844
Log_10_(TTL) = a + b * Log_10_(SVL)	0.41689 (0.31918, 0.51459)	0.0425	0.91905 (0.85267, 0.98543)	0.0296	0.9836
Log_10_(BM) = a + b * Log_10_(TTL)	-5.1240 (-5.76438, -4.48354)	0.3488	2.9221 (2.6513, 3.19297)	0.1496	0.9797
Log_10_(BF) = a + b * Log_10_(BM)	2.21779 (2.01402, 2.42156)	0.0942	0.66776 (0.55584, 0.77968)	0.0539	0.9380

**Table 3:** Size, mass, and bite force estimates for the specimen of *P*. *brasiliensis* studied (DCL = 1400 mm).

Total body length (TTL)	Snout-vent length (SVL)	Body mass (BM)	Bite force (BF)
1,249.9 cm (988.9–1,579.7)	824.2 cm (642.8–1,056.7 cm)	8,423.9 kg (5,613.4–12,641.6 kg)	69,039.2 N (41,274.6–115,480.4 N)

Values in parenthesis are estimates within the 95% prediction limits.

This work estimated that the *Purussaurus brasiliensis* specimen was 12.5 m long in life, weighed 8,424 kg (around 8.4 metric tons), had a daily food intake between 21.6 kg and 59.5 kg, and was capable of generating a sustained bite-force of 69,039.2 N (around 7 tons-force).

## Discussion

Regression analysis of the original data provided by [22] and [28] allowed us to estimate values of total length, snout-vent length, body mass, and bite force for *P*. *brasiliensis*, using a bootstrap procedure to compute confidence intervals and standard errors. However, these estimates should be interpreted with caution as regression analysis can usually only be used to predict values for dependent variables within the range of their observed values [34]. Even so, the estimated values for *P*. *brasiliensis* are compatible with those obtained for other crocodyliformes [43, 22].

The biometric estimates of *P*. *brasiliensis* confirm that it was an apex predator. In its paleoecosystem, it was unmatched by any other carnivore. Moreover, when compared with top predators of other geological times, such as *Tyrannosaurus rex* or *Carcharocles megalodon*, and other giant extinct crocodylomorphs such as *Deinosuchus* sp. (Late Cretaceous of the United States of America), *P*. *brasiliensis* seems to have had one of the most powerful bites among tetrapods. The actual measures also indicate that it was the largest and heaviest crocodylomorph ever recorded. Such impressive measures have many ecological implications, and may have led to changes in body structures to deal with extreme weight and forces. Some of these effects are discussed presently.

### Feeding Ecology of *Purussaurus brasiliensis*

The extreme size and power reached by *P*. *brasiliensis* may be an adaptive response to competition, which occurs naturally to avoid resource-use overlap. A common feature claimed for animal guilds that appear to segregate strongly along a resource dimension is that adjacent species tend to exhibit differences in body size or feeding structures [44, 45]. That is seen in Solimões Formation crocodilians, which diverge markedly in size and overall cranial structure (e.g. *Mourasuchus*, *Gryposuchus* and the different types of Caimaninae). Eusuchians are morphologically conservative in their postcranium, varying mostly in the skull morphology and size [46, 47] in response to dietary specializations or ontogeny [48, 49]. *Purussaurus brasiliensis* seems to have obtained its ecological segregation by substantial body enlargement and cranial specialization. This body enlargement permitted it to include a wider range of prey to its diet, and the bite force increased as the body size enlarged.

Previous authors have observed that body length is a key factor in crocodilian feeding ecology and that the proportional occurrence of different categories of food taken by crocodilians increased in relation to their length [26]. The feeding behavior of some extant caimans, as well as their size, varies ontogenetically. Younger—or smaller—individuals tend to feed mostly on insects, mollusks and fish. As they reach maturity, larger caimans modify their diet to include snakes, turtles, mammals and birds [50, 51]. Both the change in diet and increase in the size of prey point to a possible intraspecific niche-differentiation in time. In the case of *P*. *brasiliensis*, making a parallel to modern Caimaninae would suggest that as they reached gigantic sizes, their diet could include larger prey. Nevertheless, while at similar sizes, their diet was likely very similar to that of extant caimans.

A broader head in crocodilians allows the capture of larger prey [52]. Brevirostrine forms (short- and broad-snouted), which include *Purussaurus*, are usually known to rely on larger food items, which could comprise other reptiles and terrestrial mammals, while long-snouted forms include more fish in their diet [53]. Large Nile crocodiles are known to prey on large mammals, and they are the biggest extant crocodilians capable of taking animals larger than themselves, including adult African buffalo [54]. Large to very large *Crocodylus niloticus* (with a length of more than 3 m) can subdue mammals up to 900kg [26]. The size and bite force of *P*. *brasiliensis* should have allowed it to capture prey over 1 ton, if they were available.

**Fig 5:**
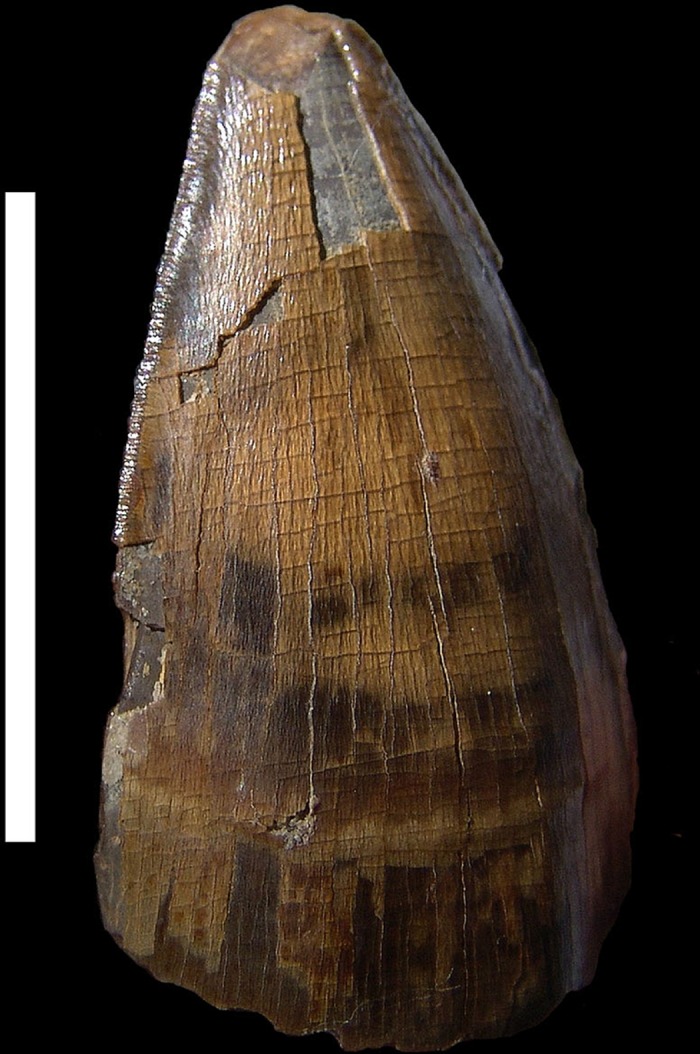
*Purussaurus brasiliensis* tooth collected at the “Cachoeira do Bandeira” site. Scale bar: 3 cm.

The teeth of *Purussaurus* sp. are subcircular at their base, slightly flattened at the crown, and bear pseudoziphodont ridges (*sensu* [55]). A gradual transition can also be seen from taller and acutely pointed anterior teeth to broader and lower posterior ones, which are more button-shaped (heterodont in shape and size). The teeth of *P*. *neivensis* have been described as curving backwards and slightly inwards [56]. Isolated teeth attributed to *P*. *brasiliensis* [19] show the same characteristics (see Fig. 5). This tooth morphology is ideal for piercing and smashing [57] and indicates a high resistance to bending forces and breakage (potentially against hard materials such as bone). On the other hand, the false ziphodont carinae, analogous to the true ziphodont morphology, assisted the teeth in puncturing and drawing through flesh [58]. All this suggests that *Purussaurus* were indeed predators of vertebrates. Due to the possession of stouter teeth, *Purussaurus* exhibited a selection towards maximizing tooth strength, also allowing in niche separation.

Rodolfo Sallas observed feeding traces in a giant unidentified turtle shell that could be attributed to *Purussaurus* (pers. comm.). Living crocodilians are known to consume turtles, and turtles have been found to make up the majority of stomach contents in some large alligators and crocodiles [26, 59, 60].

There are no other fossil records suggestive of the predation activity of *Purussaurus*, but a considerable number of large vertebrates were available as prey to *P*. *brasiliensis*. In the Upper Miocene Solimões Formation, we can identify many types of large fish and aquatic fish-eating Pelecaniform birds [3, 5]; a giant turtle (*Stupendemys souzai*) measuring more than 3.1 m in carapace length [18]; mega-herbivores, such as Caviomorpha Rodentia [61], some of which could reach up to 700 kg [62]; as well as many species of giant Xenarthra and Notoungulata [3, 63], which weighed more than a ton. These species are known to have interacted with the water surface frequently and were, therefore, likely prey. Inaccessible to other predators, this feeding niche was available solely to adult *P*. *brasiliensis*.

It is even possible that *Purussaurus* might have eaten fruits on occasion given that some authors have recorded this behavior in modern caimans [64, 65]. Several studies show plant remains increasing in frequency with an enlargement in crocodilian size [66, 67].

Our estimation of mean food intake for *P*. *brasiliensis* may be seen as a first appraisal since we have no details about the metabolic rate of this extinct taxon and do not know how similar it was to modern crocodilian species. *Purussaurus brasiliensis* and *C*. *niloticus* are both large taxa and can be considered relatively close related (Crocodylia: Brevirostres), but gigantism, as observed in *P*. *brasiliensis*, may have implications not yet fully understood in the metabolic functions of crocodilians.

If paralleled with the work of [42] in which the author observed that wild *Crocodylus niloticus* individuals ingest the equivalent of their body mass within between 129 and 160 days, an 8.4-ton *P*. *brasiliensis* ingesting an average of 22 kg to 60 kg per day could consume its own weight in food in 142 to 390 days. In this case, it is important to note that crocodilians do not need to eat frequently due to their exothermic regulatory capabilities and can survive long periods without ingesting food [68]. The stomach of adult crocodilians is more frequently found empty or nearly empty [67] since they can capture larger prey and endure a longer period without ingesting food again.

### Large body size and its evolutionary constraints

Evolving to a large body size also has some negative implications. Most of them are related to the skull structure of the animal—in order to support such massive weights and forces—and its physiology [69]. Both of them pose the large-bodied species in a delicate ecological position.

One remarkable feature of *P*. *brasiliensis* is the reduction of the nasal bones to the posterior border of a huge external nostril (45 X 32 cm), which occupies 2/3 of the rostral length (Fig. 2B). This large cranial vacuity and unique narial morphology present a vaulted palate forming a flying buttress that appears to be an adaptation to deal with massive cranial forces. We suggest that it may act as a force dissipator, allowing the rostral walls to accommodate the stress imposed by the mandibular adduction. This work also proposes that the structural modifications in the skull of *P*. *brasiliensis* suggests an adaptation to facilitate new demands caused by rostral mechanics with the stress caused by approximately 7 tons of bite-force.

Busbey [70] shows that forms with a broad snout (such as *Purussaurus*) increase the torque on the center of the rostrum because it increases the moment of the jaw margins with respect to the skull center, and that force components caused by vertical loading near the largest alveoli tend to push the nasals together. Busbey [70] also pointed out that in the nasals, the primary forces are parallel to midline, indicating that an antero-posterior shear component might exist during compressive bending. In a broader comparative context, the same author mentioned that the vaulted palate occurring in high and compressed (oreinirostral) skull forms, such as *Pristichampus* and baurusuchids, form “a sort of internal flying buttress” that would help dissipate vertical forces along the tooth rows. *Purussaurus brasiliensis* (and *P*. *mirandai*) show a deeply concave dorsal surface of the frontals and pre-frontals, just in the antorbital sector of the rostrum, which [70] adverted to be the anchor region receiving compressive forces. This morphology is a “reverse” reminiscent of that flying buttress noted in the palate of oreinirostral forms, suggesting that it also acted as a stress dissipator. The retraction of nasals close to this vaulted region, and consequent enlargement of the external naris, seem to be secondary consequences of this arched rostral morphology, also contributing to eliminate the way of transmission of the antero-posterior shear component, as cited by [70].

Besides physical constraints of a large body size, such as dealing with new bending forces in the skull (in the case of carnivores) or the necessity of new specific adaptations in the general body structure for supporting weight (which is not a significant constraint for a primarily aquatic predator), limitations of reptiles’ physiology should have acted as a barrier preventing *P*. *brasiliensis* to reach even larger sizes. Body temperature regulation and ecological impositions, such as the amount of food intake, growth rates, prey availability, and population size/individual home range may be among them.

The equatorial position and the configuration of its paleoenvironment, as well as the availability of large-bodied prey and the competition with other aquatic predators in a plural macro-habitat could have triggered the evolution of large body size in *Purussaurus*. Nonetheless, it may have also led it to its vulnerability to extinction. Its maintenance must have demanded such unique environmental and ecological conditions, that the large-scale changes in the local environment (see the work of [2, 20, 63]) most likely have condemned *Purussaurus*, and other local giant crocodilians (e.g. *Gryposuchus*, *Hesperogavialis*, *Mourasuchus*, etc.), to disappearance in favor of smaller species, ecologically and physiologically more plastic [4]. In general, perturbations often have disproportionately strong negative effects on larger species (K-selected) which tend to be strong interactors in food webs [62].

Within an ecosystem, species are linked to one another via a network of interspecific interactions and fluxes of energy and matter (e.g. nutrients). Disturbances in nodes involved with keystone species and/or top predators have wide effects in networks, inducing a large-scale ecosystem regime shift [71, 72]. The loss of *P*. *brasiliensis* could have had important implications for the functional diversity of the ancient Amazonian ecosystem, triggering cascading secondary extinctions, and ultimately reshaping the whole bio-network.

## Conclusions

The estimation presented here contributes to widening the upper historical bounds of crocodylomorph bite forces. Now we have estimates to *P*. *brasiliensis* (69,039.2 N or around 7 tons-force) reinforcing the observation that crocodylomorphs evolved the strongest bites among tetrapods.

The methodology presented here to calculate body measures and BF of *P*. *brasiliensis* can be used in estimating—or even recalculating—the same measurments of other fossil Crocodylia.

*Purussaurus brasiliensis* was an apex predator unmatched by any other in its ecosystem. The evolution of a large body size granted it benefits, such as the avoidance of interspecific competition during the exploration of a specific feeding niche, but also may have led it to its vulnerability.

Body size determines a multitude of species traits that can affect the structure and dynamics of ecological networks across multiple scales of organization [71]. Under this conception, measuring body size provides a relatively simple means of summarizing a large amount of biological information embedded within an ecological system. Future food web analyses and biomass studies could use the data provided by this study.

## Supporting Information

S1 Text SVL, TTL, BF Dataset for R [28]. (DOC)

S2 Text BM, TTL, BF Dataset for R [22]. (DOC)

S3 Text Script for R. (DOC)

S1 Table SVL, TTL, DCL in *Caiman latirostris* [28]. (DOC)

S2 Table BM, TTL, BF in extant Crocodylia [22]. (DOC)
