# Correction: Morphometry, Bite-Force, and Paleobiology of the Late Miocene Caiman *Purussaurus brasiliensis*

**DOI:** 10.1371/journal.pone.0124188

**Published:** 2015-03-30

**Authors:** The PLOS ONE Staff

In the Funding section, one of the funders is listed incorrectly. The complete, correct funding information is as follows:

The publication cost was fully funded by FAPEMIG and the Graduate Program in Ecology and Conservation of Natural Resources, Federal University of Uberlândia. The links are: http://www.fapemig.br/ and http://www.portal.ib.ufu.br/. The funders had no role in study design, data collection and analysis, decision to publish, or preparation of the manuscript.
