# Supplementary material for: Morphometry, Bite-Force, and Paleobiology of the Late Miocene Caiman Purussaurus brasiliensis
Source: PLoS One. 2015 Feb 17;10(2):e0117944. doi: 10.1371/journal.pone.0117944 (PMC4331287; doi:10.1371/journal.pone.0117944)
Supplement: S1 Text — (DOC) [file pone.0117944.s001.doc]

**Text S1. SVL, TTL, BF Dataset for R [28].**

| | SVL cm,"TTL cm","DCL mm" | | | --- | --- | | 28.00,56.90,66.40 |  | | 27.00,53.10,67.00 |  | | 35.30,70.00,83.20 |  | | 31.50,64.00,74.80 |  | | 29.90,58.00,71.90 |  | | 36.10,71.80,83.20 |  | | 30.30,58.40,73.00 |  | | 25.50,51.00,63.40 |  | | 27.80,52.50,66.90 |  | | 22.70,47.70,55.40 |  | | 21.40,44.40,52.80 |  | | 21.80,45.40,53.80 |  | | 24.60,50.10,57.10 |  | | 40.20,80.70,86.30 |  | | 26.20,53.70,59.80 |  | | 22.80,46.50,55. |  | | 25.40,51.80,58.90 |  | | 22.60,46.40,52.50 |  | | 23.20,45.00,53.90 |  | | 33.80,67.20,70.80 |  | | 24.50,51.00,59.80 |  | | 32.50,57.00,76.40 |  | | 32.50,59.50,77.70 |  | | 34.00,67.50,81.90 |  | | 34.00,65.00,77.90 |  | | 47.00,90.50,105.70 |  | | 31.00,62.70,75.00 |  | | 36.70,71.00,82.50 |  | | 45.50,92.00,98.90 |  | |  |  |
| --- | --- | --- | --- | --- | --- | --- | --- | --- | --- | --- | --- | --- | --- | --- | --- | --- | --- | --- | --- | --- | --- | --- | --- | --- | --- | --- | --- | --- | --- | --- | --- | --- | --- | --- | --- | --- | --- | --- | --- | --- | --- | --- | --- | --- | --- | --- | --- | --- | --- | --- | --- | --- | --- | --- | --- | --- | --- | --- | --- | --- | --- | --- |
