# Supplementary material for: Morphometry, Bite-Force, and Paleobiology of the Late Miocene Caiman Purussaurus brasiliensis
Source: PLoS One. 2015 Feb 17;10(2):e0117944. doi: 10.1371/journal.pone.0117944 (PMC4331287; doi:10.1371/journal.pone.0117944)
Supplement: S2 Text — (DOC) [file pone.0117944.s002.doc]

**Text S2. BM, TTL, BF Dataset for R [22].**

| TAXON,"BM","TTL","BF" | |  | |  |
| --- | --- | --- | --- | --- |
| Crocodylus acutus,132,294,3999 | |  | |  |
| C. intermedius,182,340,6276 | |  | |  |
| C. johnsoni,20,167,1292 | |  | |  |
| C. mindorendis,69,244,2736 | |  | |  |
| C. moreletti,110,284,4399 | |  | |  |
| C. niloticus,86,250,3043 | |  | |  |
| C. novaeguineae,154,303,5360 | |  | |  |
| C. palustris,207,332,7295 | |  | |  |
| C. porosus,272,344,8983 | |  | |  |
| C. phombifer,52,214,2107 | |  | |  |
| C. siamensis,69,238,3415 | |  | |  |
| Mecistops cataphractus,67,247,2082 | | | |  |
| Osteolamus tetrapsis,17,147,1787 | | | |  |
| Gavialis gangeticus,112,326,1895 | | | |  |
| Tomistoma schlegelii,142,347,3397 | | | |  |
| Alligator mississippiensis,142,285,5117 | | | |  |
| A. sinensis,14,150,1084 | |  | |  |
| Caiman crocodilus,20,166,1215 | |  | |  |
| C. latirostris,30,167,1467 | |  | |  |
| C. yacare,18,162,971 |  | |  |  |
| Melanosuchus niger,59,246,2696 | | | |  |
| Paleosuchus palpebrosus,13,133,900 | | | |  |
| P. trigonatus,22,150,1082 | |  | | |
