# Supplementary material for: Morphometry, Bite-Force, and Paleobiology of the Late Miocene Caiman Purussaurus brasiliensis
Source: PLoS One. 2015 Feb 17;10(2):e0117944. doi: 10.1371/journal.pone.0117944 (PMC4331287; doi:10.1371/journal.pone.0117944)
Supplement: S3 Text — (DOC) [file pone.0117944.s003.doc]

**Text S3. Script for R.**

| | #==============================================================================#  # R script to compute boostrap estimates of confidence intervals and #  # standard errors of linear regression slope and intercept of crocodylian #  # data for the paper "Morphometry, Bite-Force, and Paleobiology of the Late #  # Miocene Caiman Purussaurus brasiliensis" (submitted to PLoS ONE) # #  # #  # written by: Mauro J. Cavalcanti (maurobio@gmail.com) #  #==============================================================================#  # Initialize random number generator  set.seed(12345)  # Load required libraries  library(car) # for regression diagnostics  library(boot) # for computing bootstrap estimates  library(simpleboot) # for plotting bootstrapped scatterplots  # generic function to obtain regression coefficients (required by boot function)  regr <- function(formula, data, indices) {  d <- data[indices,] # allows boot to select sample  fit <- lm(formula, data=d)  return(coef(fit))  }  # Set working directory  #setwd("~/My Documents/Documentos/Originais/Purusaurus")  ###############################################################################  #-------- FIRST PART: Regression analysis of Caiman data from Verdade (2000) #  ###############################################################################  data2000 <- read.csv("Verdade_2000_SVLxTTLxDCL.csv")  # Transform data to logarithms  logdata2000 <- data.frame(log10(data2000$SVL), log10(data2000$TTL), log10(data2000$DCL))  # Rename columns of the logged dataframes  names(logdata2000)[1] <- "LOGSVL"  names(logdata2000)[2] <- "LOGTTL"  names(logdata2000)[3] <- "LOGDCL"  # Make all variables available by name (but this is BAD programming!)  attach(logdata2000)  # Perform OLS on the original data  print("Regression analysis of the original data from Verdade (2000):")  ols2000a <- lm(LOGSVL ~ LOGDCL, data=logdata2000)  print(summary(ols2000a))  ols2000b <- lm(LOGTTL ~ LOGSVL, data=logdata2000)  print(summary(ols2000b))  # Extract confidence intervals for the regression parameters  print("Confidence intervals for the original data from Verdade (2000):")  ci2000a <- confint(ols2000a)  print(ci2000a)  ci2000b <- confint(ols2000b)  print(ci2000b)  # Bootstrap 95% CI for regression coefficients  # bootstrapping with 1000 replications (SVL x DCL)  results2000a <- boot(data=logdata2000, statistic=regr,  R=1000, formula=LOGSVL~LOGDCL)  # view results  print("Boostrap results:")  print(results2000a)  #plot(results2000a, index=1) # intercept  plot(results2000a, index=2) # variable  # Get bootstrapped regression coefficients  coef2000a <- apply(results2000a$t, 2, mean)  print("Bootstrapped regression coefficients for data from Verdade (2000):")  print(coef2000a)  # bootstrapping with 1000 replications (TTL x SVL)  results2000b <- boot(data=logdata2000, statistic=regr,  R=1000, formula=LOGTTL~LOGSVL)  # view results  print(results2000b)  #plot(results2000b, index=1) # intercept  plot(results2000b, index=2) # variable  # Get bootstrapped regression coefficients  coef2000b <- apply(results2000b$t, 2, mean)  print("Bootstrapped regression coefficients for data from Verdade (2000):")  print(coef2000b)  # bootstrap again with simpleboot to get confidence bands  lboot2000a <- lm.boot(ols2000a, R=1000)  lboot2000b <- lm.boot(ols2000b, R=1000)  # Plot data and regression line with confidence bandes  png(file="SVLxDCL.png", height=480, width=(2*480))  plot(lboot2000a, xlab=expression('Log'[10]*'(SVL)'), ylab=expression('Log'[10]*'(DCL)'), pch=16)  dev.off()  png(file="TTLxSVL.png", height=480, width=(2*480))  plot(lboot2000b, xlab=expression('Log'[10]*'(TTL)'), ylab=expression('Log'[10]*'(SVL)'), pch=16)  dev.off()  # Estimate values for P. brasiliensis  print("Estimates for P. brasiliensis:")  dcl <- 1400  newdata2000a <- data.frame(LOGDCL = log10(dcl))  predsvl <- predict(ols2000a, newdata2000a, interval="predict")  svl <- 10 ^ predsvl[1]  minsvl <- 10 ^ predsvl[2]  maxsvl <- 10 ^ predsvl[3]  print("SVL=")  print(svl)  print("min. SVL=")  print(minsvl)  print("max. SVL=")  print(maxsvl)  newdata2000b <- data.frame(LOGSVL = predsvl[1])  predttl <- predict(ols2000b, newdata2000b, interval="predict")  ttl <- 10 ^ predttl[1]  minttl <- 10 ^ predttl[2]  maxttl <- 10 ^predttl[3]  print("TTL=")  print(ttl)  print("min. TTL=")  print(minttl)  print("max. TTL=")  print(maxttl)  detach(logdata2000)  #--- END OF FIRST PART  ###############################################################################  #-------- SECOND PART: Regression analysis of crocodylian #  ###############################################################################  # Read in data  data2012 <- read.csv("Erickson_et_al_2012_TTLxBMxBF.csv")  # Transform data to logarithms  logdata2012 <- data.frame(log10(data2012$BM), log10(data2012$TTL), log10(data2012$BF))  # Rename columns of the logged dataframes  names(logdata2012)[1] <- "LOGBM"  names(logdata2012)[2] <- "LOGTTL"  names(logdata2012)[3] <- "LOGBF"  # Make all variables available by name (but this is BAD programming!)  attach(logdata2012)  # Perform OLS on the original data  print("Regression analysis of the original data from Erickson et al. (2012)")  ols2012a <- lm(LOGBM ~ LOGTTL, data=logdata2012)  print(summary(ols2012a))  ols2012b <- lm(LOGBF ~ LOGBM, data=logdata2012)  print(summary(ols2012b))  # Extract confidence intervals for the regression parameters  print("Confidence intervals for the original data from Erickson et al. (2012)")  ci2012a <- confint(ols2012a)  print(ci2012a)  ci2012b <- confint(ols2012b)  print(ci2012b)  # Plot influential observations  influencePlot(ols2012a, main="Influence Plot (LOGBM)")  influencePlot(ols2012b, main="Influence Plot (LOGBF)")  # Bootstrap 95% CI for regression coefficients  # bootstrapping with 1000 replications (TTL x BM)  results2012a <- boot(data=logdata2012, statistic=regr,  R=1000, formula=LOGBM~LOGTTL)  # view results  print(results2012a)  #plot(results2012a, index=1) # intercept  plot(results2012a, index=2) # variable  # Get bootstrapped regression coefficients  coef2012a <- apply(results2012a$t, 2, mean)  print("Bootstrapped regression coefficients for data from Erickson et al. (2012):")  print(coef2012a)  # bootstrapping with 1000 replications (TTL x BM)  results2012b <- boot(data=logdata2012, statistic=regr,  R=1000, formula=LOGBF~LOGBM)  # view results  print(results2012b)  #plot(results2012b, index=1) # intercept  plot(results2012b, index=2) # variable  # Get bootstrapped regression coefficients  coef2012b <- apply(results2012b$t, 2, mean)  print("Bootstrapped regression coefficients for data from Erickson et al. (2012):")  print(coef2012b)  # bootstrap again with simpleboot to get confidence bands  lboot2012a <- lm.boot(ols2012a, R=1000)  lboot2012b <- lm.boot(ols2012b, R=1000)  # Plot data and regression line with confidence bands  png(file="BMxTTL.png", height=480, width=(2*480))  plot(lboot2012a, xlab=expression('Log'[10]*'(BM)'), ylab=expression('Log'[10]*'(TTL)'), pch=16)  dev.off()  png(file="BFxBM.png", height=480, width=(2*480))  plot(lboot2012b, xlab=expression('Log'[10]*'(BF)'), ylab=expression('Log'[10]*'(BM)'), pch=16)  dev.off()  # Estimate values for P. brasiliensis  print("Estimates for P. brasiliensis:")  logbm <- ols2012a$coef[1] + ols2012a$coef[2] * log10(ttl) #predttl[1]  n <- length(logdata2012$LOGBM)  xbar <- mean(logdata2012$LOGBM)  sdev <- sd(logdata2012$LOGBM)  sum <- sum(logdata2012$LOGBM)  sumsq <- sum(logdata2012$BM ^ 2)  serr <- summary(ols2012a)$sigma  alpha <- 0.05  tval <- qt(alpha / 2, n - 2)  num <- (logbm[[1]] - xbar) ^ 2  dem <- sumsq - 1 / n * (sum ^ 2)  pint <- tval * serr * sqrt(1 + 1 / n + num / dem)  bm <- 10 ^ logbm[[1]]  minbm <- 10 ^ (logbm[[1]] + pint)  maxbm <- 10 ^ (logbm[[1]] - pint)  print("BM=")  print(bm[[1]])  print("min. BM=")  print(minbm)  print("max. BM=")  print(maxbm)  logbf <- ols2012b$coef[1] + ols2012b$coef[2] * log10(bm)  n <- length(logdata2012$LOGBF)  xbar <- mean(logdata2012$LOGBF)  sdev <- sd(logdata2012$LOGBF)  sum <- sum(logdata2012$LOGBF)  sumsq <- sum(logdata2012$BF ^ 2)  serr <- summary(ols2012b)$sigma  alpha <- 0.05  tval <- qt(alpha / 2, n - 2)  num <- (logbf[[1]] - xbar) ^ 2  dem <- sumsq - 1 / n * (sum ^ 2)  pint <- tval * serr * sqrt(1 + 1 / n + num / dem)  bf <- 10 ^ logbf[[1]]  minbf <- 10 ^ (logbf[[1]] + pint)  maxbf <- 10 ^ (logbf[[1]] - pint)  print("BF=")  print(bf[[1]])  print("min. BF=")  print(minbf)  print("max. BF=")  print(maxbf) | | --- | |  |  |
| --- | --- | --- | --- |
