# Supplementary material for: Morphometry, Bite-Force, and Paleobiology of the Late Miocene Caiman Purussaurus brasiliensis
Source: PLoS One. 2015 Feb 17;10(2):e0117944. doi: 10.1371/journal.pone.0117944 (PMC4331287; doi:10.1371/journal.pone.0117944)
Supplement: S1 Table — (DOC) [file pone.0117944.s004.doc]

**Table S1. SVL, TTL, DCL in *Caiman latirostris* [28].**

| **SVL (cm)** | **TTL (cm)** | **DCL(mm)** |
| --- | --- | --- |
| 28,00 | 56,90 | 66,40 |
| 27,00 | 53,10 | 67,00 |
| 35,30 | 70,00 | 83,20 |
| 31,50 | 64,00 | 74,80 |
| 29,90 | 58,00 | 71,90 |
| 36,10 | 71,80 | 83,20 |
| 30,30 | 58,40 | 73,00 |
| 25,50 | 51,00 | 63,40 |
| 27,80 | 52,50 | 66,90 |
| 22,70 | 47,70 | 55,40 |
| 21,40 | 44,40 | 52,80 |
| 21,80 | 45,40 | 53,80 |
| 24,60 | 50,10 | 57,10 |
| 40,20 | 80,70 | 86,30 |
| 26,20 | 53,70 | 59,80 |
| 22,80 | 46,50 | 55,30 |
| 25,40 | 51,80 | 58,90 |
| 22,60 | 46,40 | 52,50 |
| 23,20 | 45,00 | 53,90 |
| 33,80 | 67,20 | 70,80 |
| 24,50 | 51,00 | 59,80 |
| 32,50 | 57,00 | 76,40 |
| 32,50 | 59,50 | 77,70 |
| 34,00 | 67,50 | 81,90 |
| 34,00 | 65,00 | 77,90 |
| 47,00 | 90,50 | 105,70 |
| 31,00 | 62,70 | 75,00 |
| 36,70 | 71,00 | 82,50 |
| 45,50 | 92,00 | 98,90 |
