# Supplementary material for: Morphometry, Bite-Force, and Paleobiology of the Late Miocene Caiman Purussaurus brasiliensis
Source: PLoS One. 2015 Feb 17;10(2):e0117944. doi: 10.1371/journal.pone.0117944 (PMC4331287; doi:10.1371/journal.pone.0117944)
Supplement: S2 Table — (DOC) [file pone.0117944.s005.doc]

**Table S2. BM, TTL, BF in extant Crocodylia [22].**

| **Taxon** | **Mean BM (kg)** | **Mean TTL (cm)** | **Mean BF (N)** |
| --- | --- | --- | --- |
| *Crocodylus acutus* | 132 | 294 | 3999 |
| *C. Intermedius* | 182 | 340 | 6276 |
| *C. Johnsoni* | 20 | 167 | 1292 |
| *C. Mindorendis* | 69 | 244 | 2736 |
| *C. Moreletti* | 110 | 284 | 4399 |
| *C. Niloticus* | 86 | 250 | 3043 |
| *C. Novaeguineae* | 154 | 303 | 5360 |
| *C. Palustris* | 207 | 332 | 7295 |
| *C. Porosus* | 272 | 344 | 8983 |
| *C. Rhombifer* | 52 | 214 | 2107 |
| *C. Siamensis* | 69 | 238 | 3415 |
| *Mecistops cataphractus* | 67 | 247 | 2082 |
| *Osteolamus tetrapsis* | 17 | 147 | 1787 |
| *Gavialis gangeticus* | 112 | 326 | 1895 |
| *Tomistoma schlegelii* | 142 | 347 | 3397 |
| *Alligator mississippiensis* | 142 | 285 | 5117 |
| *A. Sinensis* | 14 | 150 | 1084 |
| *Caiman crocodilus* | 20 | 166 | 1215 |
| *C. Latirostris* | 30 | 167 | 1467 |
| *C. Yacare* | 18 | 162 | 971 |
| *Melanosuchus niger* | 59 | 246 | 2696 |
| *Paleosuchus palpebrosus* | 13 | 133 | 900 |
| *P. Trigonatus* | 22 | 150 | 1082 |
